# Gallic acid exerts a protective or an anti-proliferative effect on glioma T98G cells via dose-dependent epigenetic regulation mediated by miRNAs

**DOI:** 10.3892/ijo.2015.2864

**Published:** 2015-02-02

**Authors:** ALESSANDRO PAOLINI, VALERIA CURTI, FRANCESCA PASI, GIULIANO MAZZINI, ROSANNA NANO, ENRICA CAPELLI

**Affiliations:** 1 Department of Biology and Biotechnology, Laboratory of Neuro Radio Experimental Biology, ‘Lazzaro Spallanzani’, University of Pavia, 27100 Pavia, Italy; 2 Department of Earth and Environmental Sciences, Laboratory of Immunology and Genetic Analysis, University of Pavia, 27100 Pavia, Italy; 3 IGM-CNR Histochemistry and Cytometry Section, Department of Animal Biology, University of Pavia, 27100 Pavia, Italy

**Keywords:** gallic acid, glioblastoma, cytotoxic effects, miRNAs

## Abstract

Glioblastoma multiforme (GBM) is the most malignant primary brain tumor in adulthood, characterized by very high recurrence. Following the limited results for conventional therapies, novel therapeutic agents are under investigation. Among the putative new molecules, gallic acid (GA) represents a promising new anticancer drug. The anticancer effect of this drug has been based on its antioxidant effects. The aim of the present study was to investigate the toxic effects of GA on the T98G human glioblastoma cell line and its capacity to modulate the expression of microRNAs targeting the genes involved in tumor growth and invasion. Cytotoxicity, clonogenic ability and cell migration after GA treatment were tested. Moreover, the expression of miRNAs that target genes for antioxidant mitochondrial enzymes (miR-17-3p), p-21 protein (miR-21-5p) and ATM (miR-421-5p) was determined by qRT-PCR. The results confirmed in the T98G cells the anti-proliferative effect of GA reported for other glioma cell lines and showed that the miRNA expression changes depending on GA concentrations. Different GA concentrations can determine a protective or a toxic effect on tumor cells. Thus, the key for GA to induce a specific anticancer action is to use an optimal concentration that avoids these twin effects.

## Introduction

The astrocytic tumors are a class of cancer whose treatment appears to be one of the greatest challenges in oncology. Glioblastoma multiforme (GBM) is the most common form of astrocytic tumor and is known to be particularly aggressive due to its speed in invading the adjacent brain structures (1). This tumor represents the 40% of all primary brain tumors and 78% of the malignant tumors of the central nervous system (CNS) (2). Surgical resection, current chemotherapy or/and radiotherapy have not yet produced appreciable results in order to improve the survival or the well-being of patients (3). Therefore, a number of studies are in progress to search for new drugs known to be more effective and with fewer side-effects, compared to existing chemotherapeutic. Among the putative molecules, phenolic compounds are of great interest in epidemiologic studies. These studies have demonstrated the protective effects of phenolic compounds against damage induced by exogenous and endogenous mutagens (4,5). These effects are believed to occur through the regulation of the signaling pathways, such as nuclear factor-κB (NF-κB), activator protein-1 (AP-1) or the mitogen-activated protein kinase (MAPK) (6,7). By modulating these cell signaling pathways, polyphenols activate cell death signals and induce apoptosis in precancerous or malignant cells resulting in the inhibition of cancer development or progression (8,9). However, regulation of cell signaling pathways by dietary polyphenols can also lead to cell proliferation/survival or inflammatory responses due to the increased expression of several genes. Many recent studies have shown that polyphenols are able to influence gene expression at the epigenetic level inducing the transcription of small non-coding RNAs, particularly microRNAs (miRNAs), that are able to inhibit the expression of target genes interfering with the translation of their RNA messengers (10,11). MicroRNAs are single strand non-coding RNAs ~19–25 nt in length, that are transcribed starting from intergenic and intronic sequences. They are released into the extracellular compartment by various proteins, lipids or exosomes, inducing a spread of molecular signals in biological fluids (12). Many studies have confirmed the effects of different miRNAs in various physiological processes such as proliferation, apoptosis and cell development. Consequently, a dysregulation in their expression, plays a fundamental role in the onset, progression and dissemination of malignant cells (13–16).

Gallic acid (GA) is one of the emerging polyphenol candidates for cancer treatment. It has been studied extensively and has demonstrated its ability to suppress cell viability, proliferation, invasion and angiogenesis in the human glioma cell lines (17,18). GA has been identified as both a pro-oxidant and an antioxidant agent (19). This dual role depends on the resulting GA:Fe(III) ratio (20), that comports the activity of GA or as scavengers of reactive oxygen species (ROS) or as inductor of ROS by depletion of glutathione (GHS) (21,22). Its anti-inflammatory (23), antimutagenic (24) and anticancer activities have been reported (25,26). Other studies underline the genotoxic effects of the antioxidants on human cells suggesting the use of antioxidants as attractive candidates for improved chemotherapeutic agents (27).

In the present study, we aimed to evaluate the *in vitro* effect of gallic acid (3,4,5-trihydroxybenzoic acid) on the T98G glioma cell line and to correlate the anti-proliferative and cell death effects with the expression of the miRNAs hsa-miR-17-3, hsa-miR-21-5p and hsa-miR-421-5p, already proven to be involved in the regulation of cancer cell pathways.

hsa-miR-17-3p was reported to act as a tumor suppressor both *in vitro* and *in vivo* for prostatic cancer and it has been demonstrated that this ability is due to the suppression of three critical primary mitochondrial antioxidant enzymes: manganese superoxide dismutase (MnSOD), glutathione peroxidase-2 (GPX-2) and thioredoxin reductase (TrxR2) (28,29). hsa-miR-21-5p negatively regulates the expression of p21 protein in the p53 network (30), and hsa-miR-421, is a miRNA that downregulates the ataxia telangiectasia mutated (*ATM*) gene expression inducing changes at S-phase level of cell cycle checkpoint and an increasing of sensitivity to ionizing radiation (31,32). Moreover, it is involved in the transforming growth factor β (TGF-β) pathway interfering with the *DPC4/Smad4* gene regulation (33).

## Materials and methods

### Drug preparation

Gallic acid was purchased from Sigma-Aldrich Co., and 100 mg was dissolved in 1 ml of dimethyl sulfoxide (DMSO) (PubChem CID: 679). Furthermore, the solution was diluted (1:10) with Eagle’s minimum essential medium (EMEM), subdivided into stock aliquots that and stored at −20°C. The solution was further diluted to appropriate concentrations using cell culture medium immediately before use.

### Cell culture

Human glioblastoma T98G cells were obtained from The European Collection of Cell Cultures (ECACC). These cells were maintained in EMEM containing 10% calf serum; 100 units/ml penicillin/streptomycin; 1% sodium pyruvate and 2 mM L-glutamine (Sigma-Aldrich, St. Louis, MO, USA). The cells were maintained in exponential growth as monolayers in 75-cm^2^ plastic tissue-culture flasks (Corning) and kept in a humidified atmosphere with 5% CO_2_ at 37°C.

### Mitotic activity index and clonogenic assay

To evaluate the mitotic activity index (MAI), confluent cells were harvested from flasks by trypsinization. Cells (2×10^4^) in a 2-ml complete medium were seeded in 2.5 cm diameter dishes containing 18×18-mm glass coverslip. After 24 h of incubation with serial dilution (100-1 μg/ml) of gallic acid, during the process of taking out the drug, the coverslips were reset and stained with MGG. The mitotic activity was assessed by examining ~10 consecutive high-power fields (HPFs) with an Olympus BX-41 microscope in a blind manner.

For the clonogenic assay, exponentially growing cells were seeded in 25-cm^2^ flasks (Corning) at a density of 100 cells/flask in a 5-ml culture medium. After 24 h the culture medium was removed and the medium containing GA was added. After a further 24 h, the medium was replaced with fresh culture medium and the cells were grown for 12 days, with medium renewal every 3 days. After discarding the culture medium the cells were stained with crystal violet (0.5%) for 7 min, rinsed with PBS and distilled water, then all viable colonies of >50 cells, were counted. The results were normalized with the data obtained in unexposed control and expressed as colony forming efficiency, which is the ratio of the mean number of colonies in the treated condition to the mean number of colonies in the control condition.

### Proliferation and MTT (3-(4,5-dimethylthiazol-2-yl)-2,5 diphenyltetrazolium bromide) assay

To measure the cell viability, cells were seeded onto 96-well plate at a density of 1.2×10^4^ in 0.2 ml medium/well. After a 24-h incubation, the culture medium was removed and complete medium containing serial dilution (100-1 μg/ml) of gallic acid was added. Each concentration and the control untreated cells were tested in triplicate. After 24 h of treatment, 50 μl of MTT solution was added to each well and incubated for an additional 3 h. The medium was then discarded, and the formazan crystals obtained were solubilized by adding 150 μl of DMSO. The absorption of formazan in solution was measured at the wavelength of 570 nm by an ELISA plate reader (Tecan Group Ltd., Mannedorf, Switzerland). To measure the anti-proliferative effects of gallic acid, 1×10^4^ cells were seeded onto 96-well plates and after overnight incubation were treated for 24 h with the same concentrations of drug described above. The cells were incubated for 24, 48 and 72 h. An MTT assay was performed at each time-point for three independent experiments.

### Cytofluorimetric analysis

To evaluate the different cell death pathway, cells were seeded at a density of 2.5×10^5^ in 25-cm^2^ flasks. After overnight incubation, the culture medium was removed and cells were incubated with complete medium containing serial dilutions (100-1 μg/ml) of gallic acid. After 24 h the cells were harvested by trypsinization and centrifuged for 6 min at 1,200 rpm. The cell pellet obtained was resuspended in 200 μl of binding buffer 1X (HEPES/NaOH 100 mM) with 1.4 MNaCl and 25 mM CaCl_2_ (pH 7.5). Finally, 7 μl of Annexin V and 3 μl propidium iodide (PI) were added to the cell suspension, and flow cytometric analyses were perfomed using a Partec PAS II instrument (Partec, Munster, Germany).

### Wound scratch assay

Cells were seeded at a density of 2×10^5^ in 1-ml complete medium/well, in 24-well plates. After 24 h the cells were treated with gallic acid as described above. At the end of the treatment the monolayers were scratched with a 1-ml plastic pipette tip to create a uniform wound. The wound area was then examined after 24 h by scratch under a phase-contrast microscope at ×4 magnification. Images of three random fields were taken and the cell migration ability was expressed by closure of gap distance.

### RNA extraction

Total RNA was extracted from cell pellets using the RNeasy Mini kit (Qiagen GmbH, Hilden, Germany) according to the manufacturer’s instructions. The quality of RNA was assessed by determining the RIN (TapeStation; Agilent Technologies). A quantitative RNA analysis was performed using fluorimetric methods by means of the Qubit^®^ platform (Invitrogen, Grand Island, NY, USA) using the Quant-iT RNA assay (declared assay range between 5–100 ng; sample starting concentration between 250 pg/μl and 100 ng/μl): 2 μl of RNA was added to 198 μl of working solution obtained by mixing 1 μl of Qubit™ RNA reagent to 199 μl of Qubit™ RNA buffer. The quantitation was performed following the calibration of the instrument with the Quant-iT RNA standards (0 and 10 ng/ml).

### Real-time reverse transcription-PCR (qRT-PCR)

Quantitative real-time PCR (qRT-PCR) was performed using cDNA obtained following the reverse transcription reaction with the miRCURY LNA™ Universal RT microRNA PCR kit: 4 μl of total RNA (5 ng/μl), were added to 4 μl of 5X reaction buffer, 2 μl of enzyme mix, 1 μl of synthetic spike-in and 9 μl of nuclease-free water and the reaction was performed using a MJ Mini thermal cycler (Bio-Rad Laboratories, Hercules, CA, USA) for one reaction cycle at 42°C for 60 min, 95°C for 5 min and the reaction products were immediately cooled at 4°C.

To evaluate the miRNA expression, qRT-PCR reactions were performed using the Universal cDNA synthesis and SYBR^®^ Green Master Mix kits. Amplification was performed in a 10-μl reaction mixture containing 4 μl of 1:80 diluted cDNA, 5 μl of SYBR-Green Master Mix and 1 μl of specific LNA probe. miR-17-3p LNA probe (ACUGCAGUGAAGGC ACUUGUAG), miR-21-5p LNA probe (UAGCUUAUCAGAC UGAUGUUGA), miR-421-5p LNA probe (AUCAACAGAC AUUAAUUGGGCGC), provided by Exiqon, using the following reaction conditions: a first step at 95°C for 10 min, 45 amplification cycles of 95°C for 10 sec followed by a step at 60°C for 1 min. U6 small nuclear RNA (snU6) was used to normalize the expression data of miRNAs and each assay was performed in triplicate using the Eco Real-Time PCR instrument (Illumina, San Diego, CA, USA). The results were analyzed by the comparative ct method (ΔΔct method using the software package of the Eco Real-Time PCR system for the calculus of the 2^−ΔΔct^ value (34).

### Statistical analysis

Data are presented as mean and standard deviation. Statistical significance was analyzed by t-test to compare two means using the GraphPad QuickCalcs. P-values <0.05 were considered significant.

## Results

**Figure 1:**
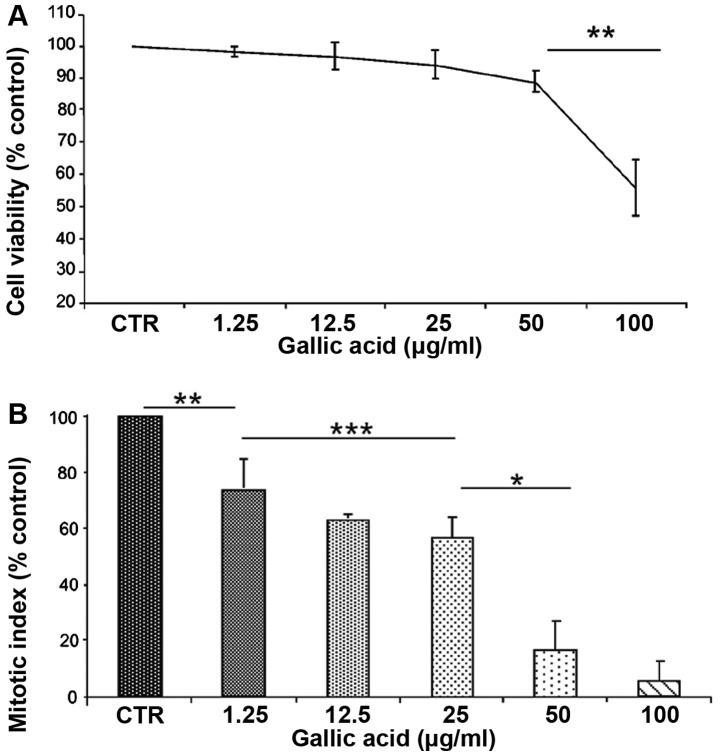
(A) Cell viability (MTT assay) and (B) Mitotic index of T98G glioma cells treated with different concentrations of gallic acid for 24 h. The results are normalized with the unexposed control (^***^P<0.05, ^**^P<0.01, ^*^P<0.001).

**Figure 2:**
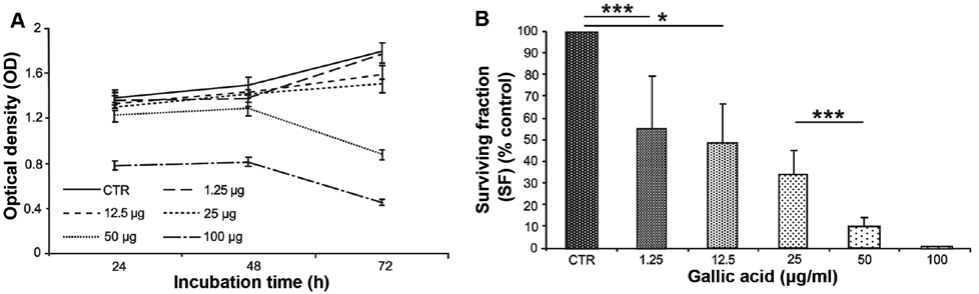
(A) Cell proliferation and (B) clonogenic ability of surviving cells. T98G cells were treated for 24 h with gallic acid and subsequently maintained in complete medium without the drug. Clone forming ability of T98G cell was assessed 12 days after the end of treatment. The results are normalized with the unexposed control (^***^P<0.05, ^*^P<0.001).

In the first phase of the investigation, the toxic effects of gallic acid were studied using MTT proliferation test, mitotic index and clonogenic assay. T98G cells were treated for 24 h with increasing concentrations of GA, ranging from 1 to 100 μg/ml. The toxic effect was evaluated determining the decrease in survival percentage (Fig. 1A) and MAI percentage (Fig. 1B) in comparison with the untreated samples. The results, summarized in Fig. 1, showed a toxic effect of GA in a concentration-dependent manner that reaches the highest value at the conditions of 50 and 100 g/ml. This highest value is correlated to a decrease in surviving cells 12 and 45%, respectively (P<0.01) and to a reduced mitotic index of 84 and 95% at the same concentrations (P<0.01). Considering cell proliferation, the maximum effect is observed after 72 h of consecutive GA treatment with a proliferation reduction of 45% at concentration of 100 μg/ml (Fig. 2A). To evaluate the capacity to repair damage induced by GA treatment, a colony forming assay was performed after the treatments. It was possible to observe that the number of colonies is progressively reduced with the increasing concentration of GA and for cells treated with the highest concentration the clonogenic capacity is completely compromised (Fig. 2B).

**Figure 3:**
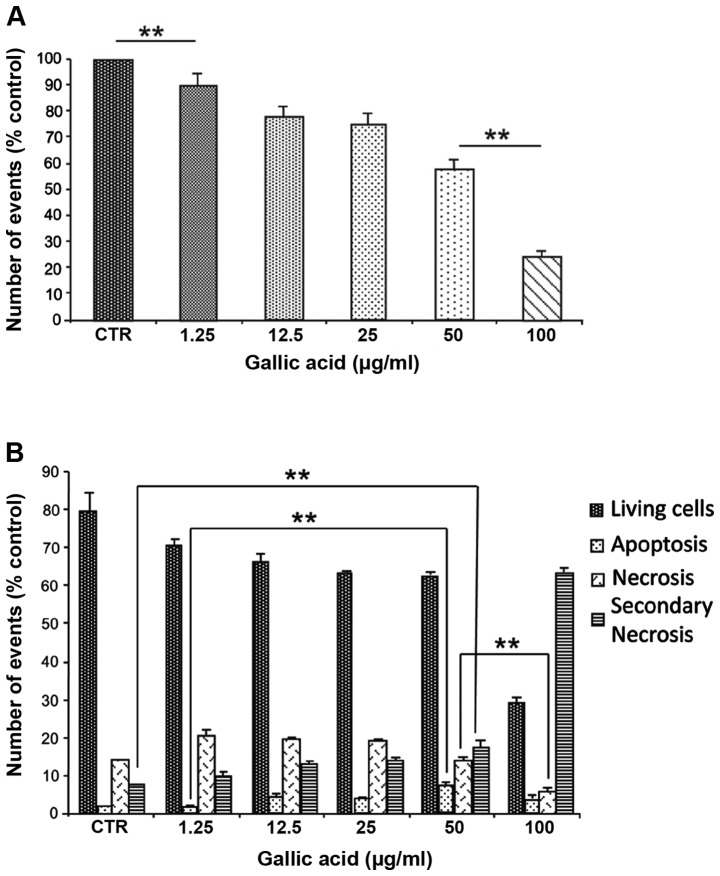
Cytofluorimetric analysis: of cells treated with GA for 24 h. Total number of events counted (A) and cell death pattern (B). Apoptotic and necrotic cells were estimated using Annexin V and PI, respectively (^***^P<0.05, ^**^P<0.01, ^*^P<0.001).

To evaluate the cell death pathway induced by GA a cytofluorimetric analysis was performed. The results indicated a progressive decrease in cell numbers with the increasing GA concentration (Fig. 3A) due to increase of necrotic (propidium iodide positive cells) and apoptosis cell fractions (Annexin V- positive cells) (Fig. 3B). At the lowest concentrations, up to 25 μg/ml, an increase in both necrotic and apoptotic cells were observed. On the contrary, at higher concentrations, the damaged cells appeared positive both to Annexin V and propidium iodide emphasizing a massive damage induced by the GA treatment (Fig. 3B).

**Figure 4:**
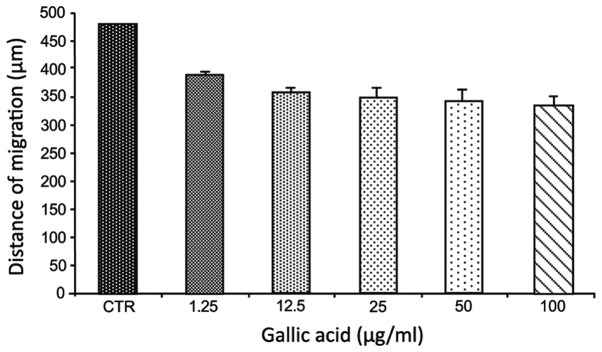
Migration ability (wound scratch assay) of T98G cells after 24 h of gallic acid treatment.

The wound scratch assay was carried out to examine the effect of GA on migration of T98G glioma cells. As compared with the control, the gap distance was not significantly reduced by gallic acid even at the highest concentration of 100 μg/ml (Fig. 4).

In the second phase of the investigation we investigated the ability of gallic acid to induce variations in the levels of hsa-miR-17-3p, hsa-miR-21-5p and hsa-miR-421. These miRNAs downregulate, respectively, the expression of three critical primary mitochondrial antioxidant enzymes (MnSOD, GPX-2 and TrxR2), the expression of the activator transcriptional factors E2F1 and E2F2 involved in cell cycle progression, and finally the expression of ATM gene involved in DNA repair.

**Figure 5:**
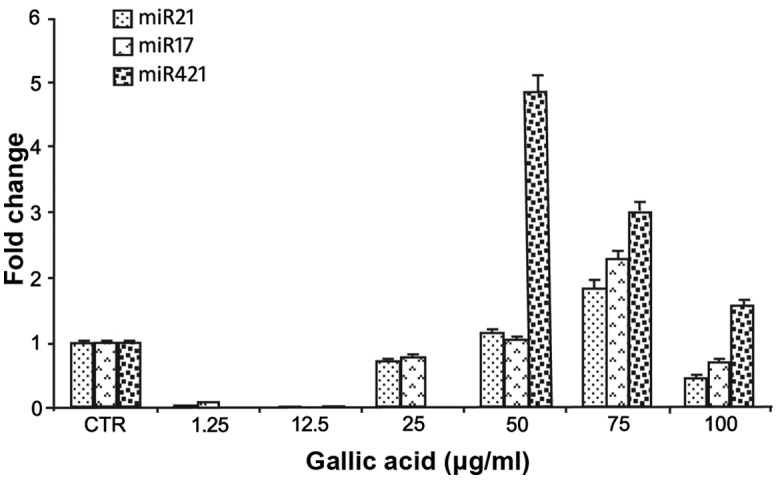
MicroRNA expression of miR-21-5p, miR-17-3p, miR-421-5p in T98G cells after treatment with gallic acid at different concentration. qRT-PCR.

The expression of these miRNAs was determined by qRT-PCR (see Materials and methods) and the results are reported in Fig. 5. The miRNAs considered showed a variation in their expression after GA treatment displaying a common reduced expression at low GA concentrations (1.2, 12.5 and 25 μg/ml) and an increased expression at concentrations >25 μg/ml (Fig. 5). The expression of the three different miRNAs seems to be modulated by different GA concentrations with a significant increase at the concentration of 75 μg/ml and a reduction at the highest concentration of 100 μg/ml (Fig. 5).

## Discussion

Our data demonstrated the anti-proliferative effects of GA on the T98G glioma cell line so confirming the anti-tumoral effects reported previously on other cell models of glioma (17). Moreover, our findings demonstrate that GA influences the expression of some miRNAs that control significant pathways involved in anti-oxidant activities, in cell cycle progression and in cell death. The effects observed are in relation with GA concentration: at low concentrations up to 25 μg/ml the expression of all the miRNA considered was reduced compared to the untreated control cells, but at concentrations >25 μg/ml a progressive induction of miRNA synthesis was observed. The reduction of miR-17 expression at low GA concentrations is indicative of an upregulation of the mitochondrial antioxidant activities and this function can be considered a beneficial effect exerted by GA as radical scavenger (20). Many epidemiological studies correlate the beneficial effects on population health to food containing polyphenols with the antioxidant property of these molecules (4,5). Following the GA concentration increase, a progressive increase of the expression levels of hsa-miR-17 occurs, which is indicative of a progressive downregulation of antioxidant activities. These results are in agreement with the toxic effects (i.e. reduction of mitotic index, increased cell death, reduced cell recovery, reduced clonogenic ability and increased apoptosis) that were significantly enhanced at concentrations of ≥50 μg/ml GA. Our results confirm recent studies which demonstrated that the tumor suppressor ability of hsa-miR-17-3p is correlated with the downregulation of the three critical primary mitochondrial antioxidant enzymes: manganese superoxide dismutase (MnSOD), glutathione peroxidase-2 (GPX-2) and thioredoxin reductase (TrxR2) (29).

We did not observe a significant effect of GA treatment on the reduction of cell migration of glioma cells. This effect could be explained with the ability of miR-17-3p to target the phosphatase and tensin homolog gene (*PTEN*) (35). *PTEN* is an oncosuppressor gene that inhibits tumor cell growth and motility by blocking the PI3K/Akt pathway. Its decrease in some malignant cancers, causes a Akt hyperactivation and the promotion of cell proliferation, migration, invasion and angiogenesis. The decrease in hsa-miR-17 levels, induced by low concentrations of GA, causes the upregulation of *PTEN* with a consequent decrease in cell migration. On the contrary, the increase in hsa-miR-17 levels, after treatment with concentrations >25 μg/ml of GA, induces the downregulation of *PTEN* and could explain the non-significant difference observed for wound scratch assay in all the conditions tested in (Fig. 4).

miR-421 regulates the cell cycle S-phase checkpoint and cellular radiosensitivity by suppressing ATM expression, a serine/threonine protein kinase that regulates DNA damage-induced at the G1-S and S phases of the cell cycle checkpoints (31). The increased expression of hsa-miR-421 downregulates *ATM* so reducing the capacity of T98G cells to repair radiation damage. This activity of GA is particularly important to control tumor cell proliferation after radiation treatments to avoid death escape of radiation-resistant cells.

hsa-miR-21-5p has been recently shown to be one of the five most abundantly expressed miRNAs in patients with colorectal cancer (36). The pathways with the most significant gene-enrichment for this miRNA belong to the ‘Pathways in cancer’, ‘Colorectal cancer’, ‘Hepatitis B’, ‘MAPK signalling pathway’, ‘Cell cycle’ and ‘Glioma’. Focusing on this latter pathway, the target genes are *E2F1*, *E2F2*, two important activator transcriptional factors, involved in cell cycle progression, in particular in the G1/S transition, that binds the proto-oncogene epithelial growth factor receptor (*EGFR*) (37). The binding of a ligand to EGFR leads to proliferation, differentiation and inhibition of apoptosis through the activation of different pathways, such as MAPK, phosphatidylinositide 3-kinases (PIK3), signal transducer and activator of transcription (STAT), cyclin-dependent kinase 6 (CDK6), PIK3R1 and *PTEN*. Additionally, hsa-miR-21-5p has been found, deregulated in pediatric cancer stem cells and in clear renal cell carcinoma (RCC) (38,39).

**Figure 6:**
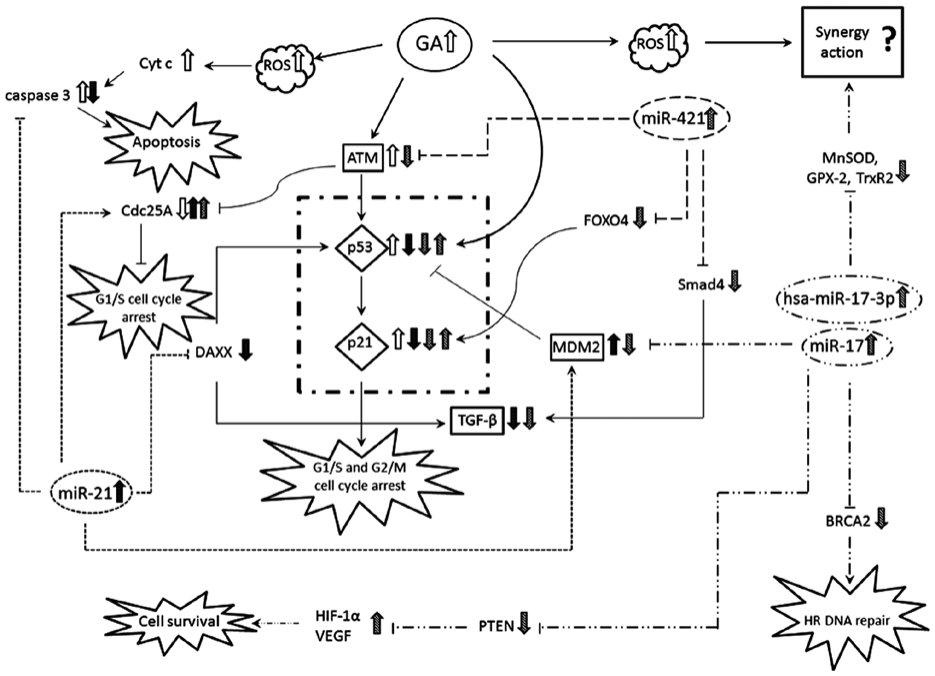
Summary diagram of the relationships between miR-21, miR-17 and miR-421 and the related targets: GA, gallic acid; ROS, reactive oxygen species; Cyt c, cytochrome *c*; CdC25A, cell division cycle 25 homolog A; DAXX, death-associated protein 6; ATM, ataxia telangiectasia mutated; p53, genome reparative protein; G1/S phase; p21, cell cycle regulator protein; TGF-β, transforming growth factor β; HIF-1α, hypoxia-inducible factor 1-α; VEGF, vascular endothelial growth factor; PTEN, phosphatase and tensin homolog; FOXO4, forkhead box protein O4; Smad4, SMAD family member 4; MnSOD, manganese superoxide dismutase; GPX-2, glutathione peroxidase 2; TrxR2, thioredoxin reductase-2; BRCA2, breast cancer 2, early onset.

Fig. 6 summarizes a possible network describing the principal mechanisms influenced by GA and the microRNAs tested. All the three microRNAs tested are involved in p53 pathway, inducing variations in expression of p21.

In conclusion, based on the present study, GA at low concentrations inhibits all the three miRNAs considered, indicating that the increase in mitochondrial antioxidant capacity (decrease of 17-3p), increases cell proliferation by stimulating the regeneration of cells and tissues (decrease of miR-21), and increases the ability to repair damage caused by chemicals and radiation (decrease of miR-421). This scenario is in agreement with the observations reported by a number of epidemiological studies that have observed a lower incidence of cancer and aging delay in populations with a diet rich in polyphenols (4,5).

Gallic acid at high doses causes a reduction in mitochondrial antioxidant activity (increased miR-17 levels); slows cell proliferation (increased miR-21 levels) and decreases the ability to repair the damage (increased miR-421 levels).

The functionality of polyphenols has been proven in numerous publications, but there are still unclear points regarding the useful concentrations. GA is a molecule that is found intact in biological fluids having a 1.2–1.5 h elimination half-life and a better absorption capacity when compared with other polyphenols (40). Moreover, it was observed that <60% of GA excreted in the urine is metabolized to its glucuroni-dated form 4-O-methylgallic acid (4OMGA) (41). Studies of the pharmacokinetics, bioavailability and toxicity on experimental models *in vivo* can provide useful information for the use of GA in the treatment or in the prevention of cancer and neurodegenerative diseases.

## Abbreviations

GBM: glioblastoma multiforme
CNS: central nervous system
NF-κB: nuclear factor-κB
AP-1: activator protein-1
MAPK: mitogen-activated protein kinases
miRNA: microRNA
GA: gallic acid
ROS: reactive oxygen species
GHS: glutathione
MnSOD: manganese soperoxide dismutase
GPX-2: glutathione peroxidase-2
TrxR2: thioredoxin reductase
DMSO: dimethyl sulfoxide
EMEM: Eagle’s minimum essential medium
ECACC: European Collection of Cell Cultures
MAI: mitotic activity index
HPFs: high-power fields
qRT-PCR: quantitative real-time PCR
PTEN: phosphatase and tensin homolog gene
EGFR: epidermal growth factor receptor
4OMGA: 4-O-methylgallic acid
ATM: ataxia telangiectasia mutated
PIK3: phosphatidylinositide 3-kinases
STAT: signal transducer and activator of transcription
TGF-β: transforming growth factor β
CDK6: cyclin-dependent kinase 6
RCC: renal cell carcinoma
